# Optical and Surface Properties of Monolithic Zirconia after Simulated Toothbrushing

**DOI:** 10.3390/ma12071158

**Published:** 2019-04-10

**Authors:** Jae-Hyun Lee, Sung-Hun Kim, Jung-Suk Han, In-Sung Luke Yeo, Hyung-In Yoon

**Affiliations:** 1 Department of Prosthodontics, One-Stop Specialty Center, Seoul National University Dental Hospital, Seoul 03080, Korea; jhlee.snudh@gmail.com; 2 Department of Prosthodontics and Dental Research Institute, School of Dentistry, Seoul National University, Seoul 03080, Korea; proshan@snu.ac.kr (J.-S.H.); pros53@snu.ac.kr (I.-S.L.Y.); drhiy226@snu.ac.kr (H.-I.Y.)

**Keywords:** dentifrice, optical property, surface roughness, toothbrushing, zirconia

## Abstract

This in vitro study investigated the impact of various dentifrices on the shade, translucency, gloss, and surface characteristics of polishing- or glazing-finished monolithic zirconia surfaces after simulated toothbrushing. Eighty square-shaped monolithic zirconia specimens were divided into two major groups based on the finishing methods—polished (P) and glazed (G). Next, specimens from the two major groups were categorized into four subgroups: stored in distilled water (DW, control); brushed with a fluoride-free conventional dentifrice (C); brushed with a fluoride dentifrice (F); and brushed with a whitening dentifrice (W). Overall, eight groups were created—PDW, PC, PF, PW, GDW, GC, GF, and GW (*n* = 10 each). Shade, translucency, surface gloss, surface roughness, crystalline phase, and superficial topography data were obtained. Repeated-measures ANOVA and two-way ANOVA were used for intergroup comparison (all *α* = 0.05). The color differences (Δ*E*_00_) between pre- and posttreatment were 0.3158 (PDW), 0.7164 (PC), 0.7498 (PF), 0.8106 (PW), 0.1953 (GDW), 0.301 (GC), 0.3051 (GF), and 0.4846 (GW). A statistically significant difference was observed among the Δ*E*_00_, surface gloss, and surface roughness of monolithic zirconia. Thus, brushing with several dentifrices markedly affects the optical properties and surface characteristics of monolithic zirconia finished with polishing or glazing methods.

## 1. Introduction

Monolithic yttria-stabilized tetragonal zirconia (Y-TZP) is a predictable dental restorative material that exhibits a high success rate in clinical practice and is more frequently selected for an aesthetic restoration as its translucency improves [1,2,3]. Compared with conventional bilayered zirconia crowns and metal-ceramic restorations, monolithic zirconia restorations have the advantage of less ceramic fracture [4]. In addition, compared with conventional metal-ceramic restorations, monolithic zirconia crowns exhibit excellent translucency; moreover, monolithic zirconia is aesthetic because of the lack of metal exposure at the restoration margin, even when gingival recession of the abutment tooth occurs [5,6]. Thus, monolithic zirconia offers several advantages as an aesthetic restorative material, rendering it the first-choice material in the premolar region based on its tooth color and intensity. Furthermore, monolithic zirconia restorations are increasingly used in the anterior teeth, owing to the development of zirconia materials with high translucency [7,8,9].

The toothbrushing procedure involves applying a mechanical force to the tooth surface over a prolonged period [10]. In addition, various dentifrices have been developed for use with toothbrushes, and chemical components and abrasives of dentifrices can chemically and physically affect the surface of brushed teeth [11,12]. The high fluoride concentration in a dentifrice has been reported to diminish the properties of dental ceramics [13,14,15]. In addition, dentifrices developed for improved tooth-whitening effects affected the optical properties of restorative ceramic materials [12,16]. Based on the abrasive content, toothpastes vary in their abrasion of enamel, as measured by the relative dentin abrasion (RDA) value [10]. Investigating toothbrushing effects on shade or translucency is important. Furthermore, it is imperative to investigate whether toothbrushing may increase the surface roughness that can result in plaque accumulation and bacterial adhesion.

Studies have reported the effects of toothbrushing and dentifrices on various restorations [12,17,18,19]; however, limited studies have investigated the impact of various dentifrices on the monolithic zirconia material. Hence, this study investigated the effects of various toothpastes on the optical properties and surface properties of monolithic zirconia finished by polishing or glazing methods. In this study, the null hypothesis is that no significant change in the optical properties and surface characteristics of polished or glazed monolithic zirconia specimens occurs after the simulated toothbrushing procedure with various dentifrices.

## 2. Materials and Methods

### 2.1. Specimen Preparation

Eighty square-shaped (22.0 mm × 22.0 mm × 2.0 mm) specimens were cut from presintered blocks of monolithic Y-TZP zirconia (Rainbow Shade Block, Shade A2; Genoss, Suwon, Korea) with a low-speed diamond disc (Diamonde Blade, Samsung Clover, Seoul, Korea) under water cooling [17]. The specimens’ thicknesses were adjusted to 2 ± 0.01 mm with a horizontal grinding machine (HRG-150; AM Technology, Asan, Korea) and were confirmed using a digital caliper (BD500-150; Bluetec, Seoul, Korea).

Coloring procedures were performed on only one side of each specimen with a metal-free coloring brush (Maedeum No. 5; Daeheung-dang, Seoul, Korea) and A3-shaded coloring liquid (Luxen CL shade A3; Dental Max, Seoul, Korea) with brushing three times to simulate the restoration coloring procedure in a dental laboratory. Then, all specimens were sintered in a furnace (PDF-1000; Dental Max, Seoul, Korea) for 10 h, including 2 h at 1530 °C as per the manufacturer’s instructions. The final dimensions of the specimens after the sintering procedure were 18.0 mm × 18.0 mm × 1.6 mm, considering approximately 20% volumetric shrinkage.

**Figure 1:**
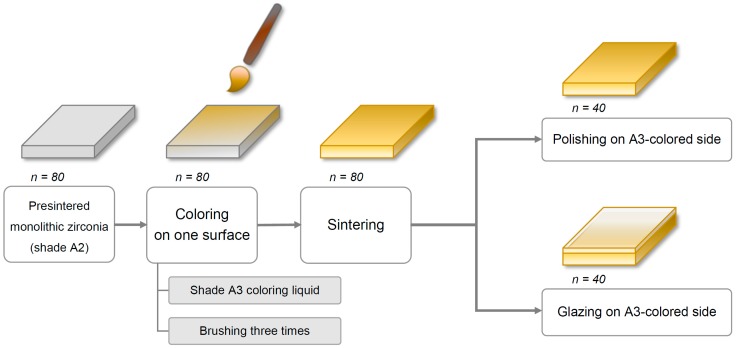
Specimen preparation.

Next, all specimens were divided into two major groups based on the finishing methods—polishing (P) and glazing (G). For glazed specimens (*n* = 40), the glazing material (Glaze HeraCeram; Heraeus Kulzer, Hanau, Germany) was coated on the A3 coloring liquid-applied surface of the specimens and fired in a ceramic furnace (Programat P310; Ivoclar Vivadent, AG, Schaan, Liechtenstein) as per the manufacturer’s guidelines. Of note, no extrinsic staining was performed. Next, the glazed surfaces of the square-shaped specimens were wet-ground with 320-, 1200-, and 2000-grit silicon carbide abrasive paper (C357; Paco Tech., Seoul, Korea), creating specimens with a glazed layer of 50 (±30) μm thickness. For polished specimens (*n* = 40), an experienced dental laboratory technician manually polished the A3 colored surface of the specimens using a zirconia polishing set (StarGloss blue/pink/gray; Edenta AG, Hauptstrasse, Switzerland) (Figure 1). Finally, a fiducial mark was engraved on the edge of the non-tested side of each specimen, which was used for distinction between groups.

Then, each finishing group was further categorized into the following four subgroups based on the brushing procedure and dentifrice used (*n* = 10/group): storage in distilled water (DW, control); brushing with a fluoride-free conventional dentifrice (C); brushing with a fluoride dentifrice (F); and brushing with a whitening dentifrice (W). Finally, based on the finishing and brushing methods, eight groups were defined as PDW, PC, PF, PW, GDW, GC, GF, and GW. Before performing tooth brushing or storage, all specimens were ultrasonically cleaned for 5 min.

### 2.2. Toothbrushing with a Dentifrice Slurry

**Figure 2:**
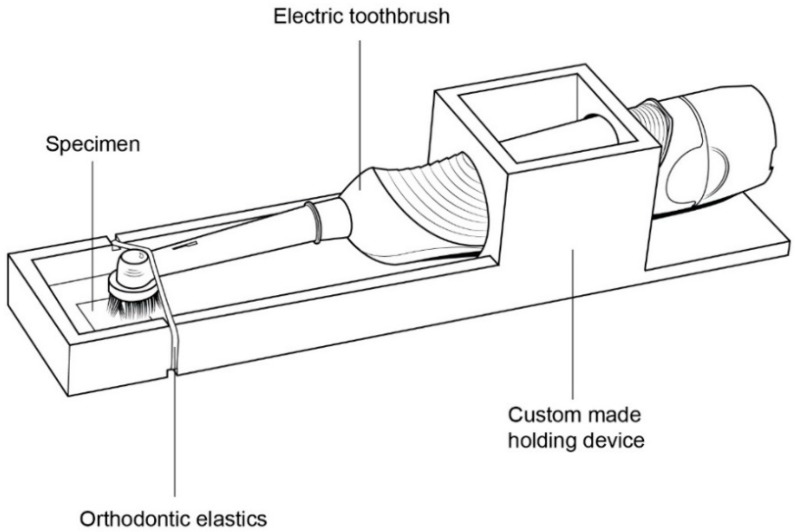
Schematic drawing of the customized fixture, zirconia specimen, orthodontic elastics, and electric toothbrush.

All specimens were subjected to a single focal area of toothbrushing using an electric toothbrush (DB-4010; Oral-B Braun GmbH, Kronberg/Ts., Germany) with a cup-shaped toothbrush head (Precision Clean; Oral-B Braun GmbH) fixed on a customized toothbrush-holding device (Figure 2). This electric toothbrush had oscillatory-rotating movement at a rate of 7600 strokes/min. The electric brushes were set to brush in “continuous mode” with a standardized vertical load of 2 N [18,19]. The vertical force was generated using orthodontic extraoral elastics 0.5-inch Extraorale Latex-Gummiringe (Dentaurum, Ispringen, Germany) and validated using a laboratory force gauge (J14002; Zeast Co., Beijing, China).

In this study, three dentifrices were used—a fluoride-free conventional dentifrice (Parodontax Classic Fluoridfrei; GlaxoSmithKline, Bühl, Germany), a fluoride dentifrice (Parodontax Fluorid; GlaxoSmithKline), and a whitening dentifrice (Crest 3d White Vivid; Procter & Gamble, Cincinnati, OH, USA). The reported RDA values for these dentifrices were 56, 56, and 233 [11]. In addition, the fluoride concentration for these dentifrices were 0, 1400, and 1500 ppm, according to the manufacturer’s guidelines (Table 1). Of note, an RDA value of 250 is the American Dental Association (ADA)–specified limit, and 1500 ppm fluoride ion in the dentifrice is the maximum concentration that can be purchased without a prescription in most countries. Each toothpaste was mixed with DW in a ratio of 1:4 to make a slurry along with the ISO (International Standards Organization) 11609:2017 standard (Dentistry-Toothpastes: Requirements, test methods and marking).

The total brushing time was calculated based on a brushing time of 120 s two times a day of all 28 teeth [10,20]. As a tooth has several surfaces to be brushed, the maximum contact time per tooth surface per day has been reported to be 5 s [10,21]. In addition, the simulated brushing time of 260 min for one surface of the specimen was evaluated to be equivalent to 8.5 years of tooth brushing.

Based on a typical toothbrush replacement cycle, bristles must be replaced after 45 days of use [10]. Reportedly, brushing all 28 teeth with 72 surfaces for 45 days is equivalent to simulated toothbrushing for 270 min, assuming that one surface is being brushed for 5 s per day [10]. Thus, in this study, the first simulated brushing was performed for 260 min (which simulated 8.5 years of toothbrushing); then, the optical properties were assessed; toothbrush heads, dentifrice slurries, and batteries were replaced; and another 260 min of brushing was performed again. Each specimen was brushed for 520 min, representing 17 years of brushing. The specimens in the DW (control) group remained submerged in DW for the same period of 520 min.

Next, 60 new electric toothbrushes and 60 new customized brush-holding devices were prepared to ensure equal experimental conditions. In addition, toothbrushing of 60 specimens was performed at the same time. After simulated brushing, all test specimens were rinsed with tap water for 30 s before all measurements.

### 2.3. Color and Translucency

To assess shade and translucency changes, the Commission Internationale de l’Éclairage (CIE) L*, a*, and b* color coordinates of 80 specimens were evaluated using a dental spectrophotometer (EasyShade V, VITA Zahnfabrik, Bad Säckingen, Germany); this device has high repeatability, with intradevice intraclass correlation coefficients (ICCs) of 0.913–0.993 [22]. In this study, each of 10 glazed zirconia specimens was measured three times to calculate the ICC and ensure the repeatability of the device used. When measuring three times, the device tip was removed from the evaluated surface of the specimen >10 cm and contacted again for other measurements to simulate a similar situation with experiments. The intradevice ICCs of the device used in this study were 1.000 for L* and a* and 0.999 for b*. To avoid the likelihood of interdevice disagreement, all measurements were made by using only one device.

The CIE L*, a*, and b* color components of each specimen were detected over white, gray, and black polytetrafluoroethylene backgrounds (GC-3, Color calibration cards; JJC Co., Seoul, Korea) at 3 different intervals—baseline, after 260 min (simulating 8.5 years), and 520 min (simulating 17 years)—of brushing. All measurements were performed by a single trained prosthodontist (JHL) under standardized D65 light illumination (18W/D65; Philips, Santiago, Chile) of the color assessment cabinet (CAC-4, Zhengzhou Hengchen Electric Tech., Henan, China). Of note, all measurements were performed with the probe tip perpendicular to the center of the specimens. In addition, the spectrophotometer was calibrated according to the manufacturer’s instructions before each color measurement to minimize the measurement uncertainty. Furthermore, the measurements for each background of each specimen were performed four times, and the mean of four measurements was recorded for data analysis.

Measurements acquired on the gray background were used to evaluate the color difference between before and after brushing. Furthermore, CIEDE2000 color differences (Δ*E*_00_) in each group between the baseline and simulated 8.5 years and between the baseline and simulated 17 years of toothbrushing were determined using the following formula [23,24]:

(1) $$\Delta E_{00}=\sqrt{{\left(\frac{\Delta L^{\prime }}{K_{L}S_{L}}\right)}^{2}+{\left(\frac{\Delta C^{\prime }}{K_{C}S_{C}}\right)}^{2}+{\left(\frac{\Delta H^{\prime }}{K_{H}S_{H}}\right)}^{2}+R_{T}\left(\frac{\Delta C^{\prime }}{K_{C}S_{C}}\right)\left(\frac{\Delta H^{\prime }}{K_{H}S_{H}}\right)}$$

where Δ*L*′, Δ*C*′, and Δ*H*′ are the differences in lightness, chroma, and hue; *S_L_*, *S_C_*, and *S_H_* are weighting functions; and *R_T_* is a rotation factor [24]. In this study, *K_L_*, *K_C_*, and *K_H_* are parametric factors set to 1.

Furthermore, the CIE L*, a*, and b* measurements acquired on the white and black backgrounds were used to evaluate the translucency parameter (TP) by estimating the CIEDE2000 color difference (Δ*E*_00_) between the color values obtained against white and black backgrounds at each test period [25].

### 2.4. Surface Gloss

After completing the entire brushing process, the surface gloss was measured three times using a small area glossmeter (WG60; FRU, Beijing, China) at the center of each sample, and the average was recorded. In specific, all specimens were placed in a black opaque container and then covered with the glossmeter to eliminate external light exposure and hold the correct position during the examination. Notably, the glossmeter was calibrated before each measurement. The projection angle of the glossmeter was 60°, and the measurement range was from 0 (for a totally nonreflective surface) to 200 (for a totally reflective surface) gloss units (GU). The glossmeter was designed and manufactured with reference to the international standard ISO 2813.

### 2.5. Surface Roughness

The surface roughness was measured on each brushed surface after all interventions with simulated cycles using a Zeiss laser scanning microscope (LSM) 800 MAT confocal scanning laser system combined with a Zeiss Axio imager Z2m microscope with ZEN software (Zeiss, Jena, Germany). On the LSM 800 MAT, imaging was made using laser excitation at 405 nm with a C Epiplan-APOCHROMAT 20 × 0.7 NA. The images were acquired at three sites within the area where each sample was brushed, and the mean of three Ra values was documented. Ra is the arithmetical mean deviation, and the measurements were made with reference to the international standard ISO 4287.

### 2.6. X-ray Diffraction (XRD)

After completing all the brushing cycles, one randomly selected sample from each subgroup was subjected to XRD (D8 Advance; Bruker, Karlsruhe, Germany) using Cu-Kα radiation (*λ* = 1.5406 Å) to ascertain the crystalline phase of each zirconia specimen. The scan was performed at a step size of 0.02° with a scan rate of 2°/min in the 2-theta range between 20° and 60°.

### 2.7. Scanning Electron Microscopy (SEM)

One representative test specimen in each subgroup was selected for SEM (Model S-4700 SEM; Hitachi High-Technologies Co, Tokyo, Japan) examination after all interventions with simulated brushing cycles. The specimens were sputtered with platinum (Q150T Sputter Coater; Quorum Technologies Ltd., Ashford, Kent, UK) and photographed at an acceleration voltage of 15 kV at magnifications of ×1000 and ×5000.

### 2.8. Statistical Analysis

All statistical analyses in this study were performed using IBM SPSS Statistics (v24.0; IBM Corp., Chicago, IL, USA). Repeated-measures analysis of variance (ANOVA) was performed to analyze Δ*E*_00_ and TP (*α* = 0.05) with brushing time as a repeated factor and toothbrushing groups as a fixed factor. Separate analyses were conducted for each dependent variable, and a Bonferroni correction was performed. In addition, two-way ANOVA was used to determine the effects of two factors, the finishing methods and dentifrices used, on the Δ*E*_00_, TP, GU, and Ra outcome variables. The interactions between the two factors were also analyzed. In this study, the statistical significance was set at 0.05 for all analyses.

## 3. Results

### 3.1. Color and Translucency

**Figure 3:**
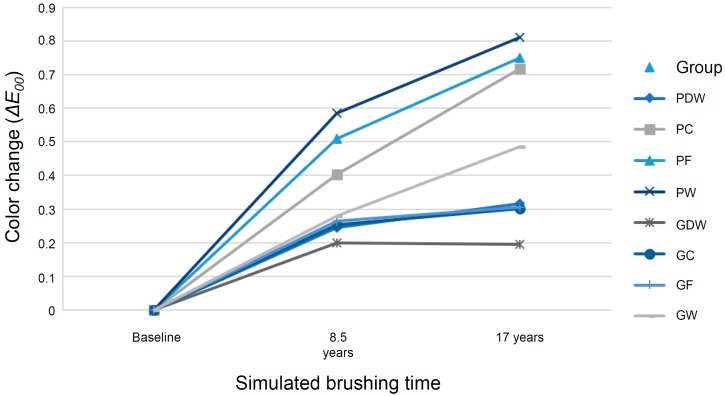
Color changes (Δ*E*_00_) of polished or glazed monolithic zirconia specimens between the baseline and simulated brushing time.

Table 2 summarizes the results of the repeated-measures ANOVA of color change and TP. Of note, analyses were performed separately for color changes and TPs. Because the color changes as the dependent variable did not satisfy a sphericity assumption (*p* = 0.002) of the repeated-measures ANOVA, the Greenhouse–Geisser assumption (*p* = 0.857) was used. The repeated-measures ANOVA revealed a significant impact of simulated years and groups on color differences (Δ*E*_00_; *p* < 0.001). Table 3 summarizes the mean and standard deviation values of color change for each group during the simulated 17 years of toothbrushing. Analyses were performed separately according to the finishing methods—polished and glazed. In the polishing-finished groups, the brushed groups displayed significantly more color changes than PDW. In the glazing-finished groups, GW exhibited greater shade change than GDW (*p* = 0.014; Figure 3). Table 3 and Figure 3 show that the Δ*E*_00_ values of most groups, except PW, were within the 50%:50% perceptibility threshold based on previous studies (0.80–1.30 Δ*E*_00_ units) [26,27,28]. However, the Δ*E*_00_ of PW was still within the clinically acceptable color change threshold (1.80–2.25 Δ*E*_00_ units) [26,27,28,29].

**Figure 4:**
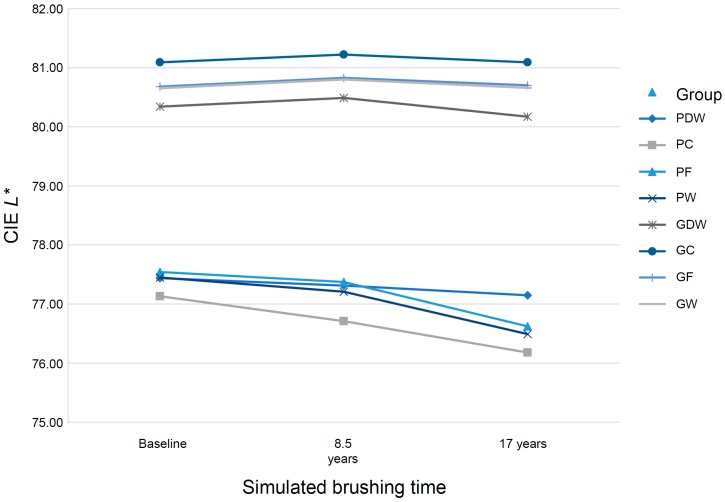
CIE L* of polished or glazed monolithic zirconia specimens according to simulated brushing time.

In this study, repeated-measures ANOVA was performed for each of the CIE L*, a*, and b* values. The CIE L*, a*, and b* color coordinates exhibited significant differences in time and time × group interaction, respectively. A significant tendency in L* was observed, but in the glazed groups, there was no significant difference in the L* value with time. However, in the polished groups, a marked decline in the L* value after brushing was observed, indicating that the specimen was darkened. In the polished groups that were brushed, a higher decline in the L* value than that in PDW was observed (Figure 4).

Furthermore, two-way ANOVA revealed marked differences in color changes based on finishing methods and dentifrice used (Table 4 and Table 5). The polishing-finished groups exhibited significantly higher color change values than the glazing-finished groups (*p* < 0.001).

In this study, TP satisfied a sphericity assumption of the repeated-measures ANOVA; however, no significant change was shown after simulated toothbrushing, irrespective of the period and experimental group (*p* > 0.05; Table 2). Table 6 presents the mean values and standard deviations of TP during the experimental interventions. The two-way ANOVA for TP exhibited no marked difference based on the finishing method and dentifrice used (Table 4 and Table 5).

### 3.2. Surface Gloss

Two-way ANOVA revealed that the finishing methods (*p* < 0.001) and dentifrice used (*p* = 0.005) exerted a marked impact on the surface gloss (Table 4); however, no significant interaction was found between the finishing method and dentifrice (*p* = 0.874). Table 5 shows that groups brushed with a whitening dentifrice exhibited a lower surface gloss value than the groups brushed with a conventional dentifrice and stored in DW after 17 years of simulated toothbrushing. Furthermore, the glazing-finished groups exhibited markedly lower GU than the polishing-finished groups after all interventions. Table 7 presents the means and standard deviations of GU in each group.

### 3.3. Surface Roughness

**Figure 5:**
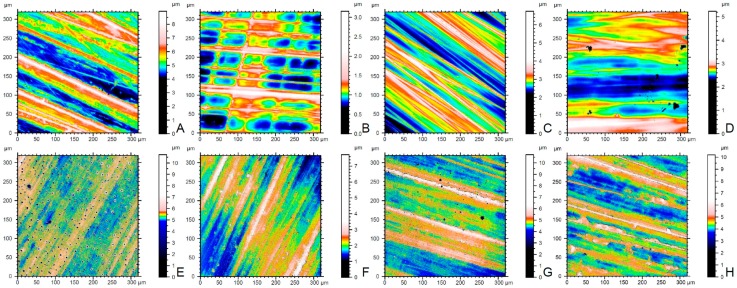
Confocal laser scanning microscope images of groups (fields of view: 320 µm × 320 µm). (**A**), polished surface and storage in distilled water (PDW). (**B**), polished surface and brushed with a conventional dentifrice (PC). (**C**), polished surface and brushed with a fluoride dentifrice (PF). (**D**), polished surface and brushed with a whitening dentifrice (PW). (**E**), glazed surface and storage in distilled water (GDW). (**F**), glazed surface and brushed with a conventional dentifrice (GC). (**G**), glazed surface and brushed with a fluoride dentifrice (GF). (**H**), glazed surface and brushed with a whitening dentifrice (GW).

Table 4 presents no significant interaction between the specimen finishing method and dentifrice used based on two-way ANOVA (*p* = 0.123). The finishing methods (*p* < 0.001) and dentifrices (*p* = 0.048) each markedly affected the surface roughness of the tested specimens. The glazing-finished groups presented a rougher surface than the polishing-finished groups after all interventions (Table 5). In addition, GF exhibited significantly higher Ra values than GDW (*p* = 0.004; Table 7). Figure 5 displays representative surface images obtained by a confocal laser scanning microscope.

### 3.4. X-ray Diffraction (XRD)

**Figure 6:**
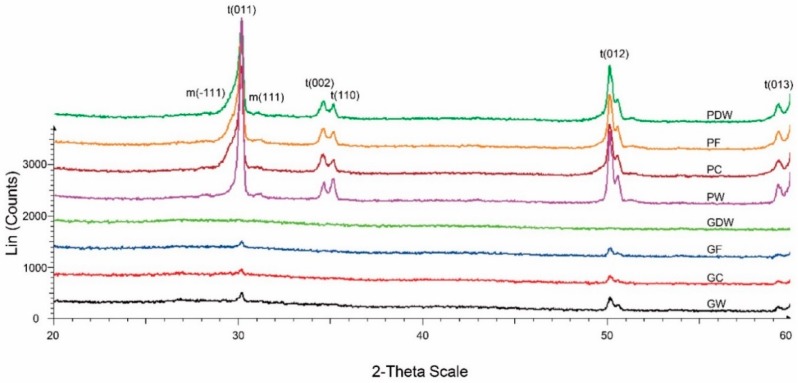
X-ray diffraction patterns of experimental groups in the 2-theta range from 20 to 60. t, Tetragonal zirconia phase; m, monoclinic zirconia phase; PDW, polished surface and storage in distilled water; PC, polished surface and brushed with a conventional dentifrice; PF, polished surface and brushed with a fluoride dentifrice; PW, polished surface and brushed with a whitening dentifrice; GDW, glazed surface and storage in distilled water; GC, glazed surface and brushed with a conventional dentifrice; GF, glazed surface and brushed with a fluoride dentifrice; GW, glazed surface and brushed with a whitening dentifrice.

In this study, monoclinic peaks were rarely detected in all groups (Figure 6). The polishing-finished groups (P line groups) exhibited similar crystallographic patterns. Comparatively, specimens covered with glazing material (G line groups) exhibited a weaker signal. Furthermore, compared with GDW, which exhibited no high peaks, GC, GF, and GW, which were G line groups that were brushed, exhibited several high tetragonal peaks.

### 3.5. Scanning Electron Microscopy (SEM)

**Figure 7:**
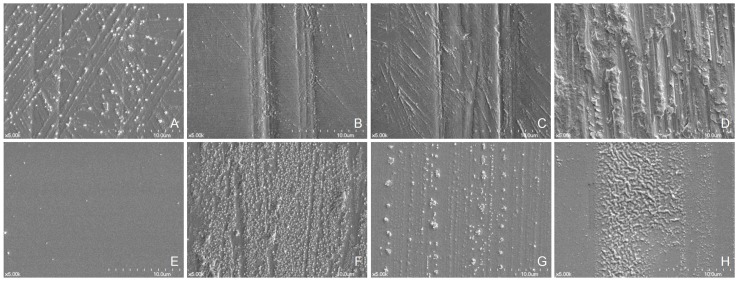
Scanning electron microscopy (SEM) images of groups (original magnification, ×5000). (**A**), polished surface and storage in distilled water (PDW). (**B**), polished surface and brushed with a conventional dentifrice (PC). (**C**), polished surface and brushed with a fluoride dentifrice (PF). (**D**), polished surface and brushed with a whitening dentifrice (PW). (**E**), glazed surface and storage in distilled water (GDW). (**F**), glazed surface and brushed with a conventional dentifrice (GC). (**G**), glazed surface and brushed with a fluoride dentifrice (GF). (**H**), glazed surface and brushed with a whitening dentifrice (GW).

Figure 7 displays SEM images (magnification, ×5000) of specimens exhibiting differences in surfaces. The surfaces of the brushed groups (PC, PF, PW, GC, GF, and GW) exhibited scratches and striated patterns caused by toothbrushing procedures. In addition, striated patterns, which were created by manually controlled polishing instruments, were observed on the surfaces of PDW specimens. Conversely, the surfaces of the GDW specimens revealed no wear tracks of abrasion.

## 4. Discussion

This study evaluated the impact of toothbrushing on the optical properties and surface characteristics of monolithic zirconia materials. The findings rejected the null hypothesis for both optical properties and surface roughness. Statistically significant changes in color parameters were observed as toothbrushing progressed, and a decline in the surface gloss of the groups brushed with fluoride and whitening dentifrices compared with the group stored in DW was identified. In addition, the surface roughness of the glazed group brushed with a fluoride dentifrice appeared markedly rougher than the unbrushed glazed group. After toothbrushing, the glazed groups exhibited markedly higher color stability than the polished groups; however, the glazed groups exhibited less surface gloss and rougher surfaces than the polished groups.

Our findings corroborate a previous study on color change in brushed zirconia specimens. Yuan et al. [30] reported a statistically significant shade change in extrinsically stained and glazed zirconia specimens after 15 years of simulated brushing. While the evaluated Δ*E* value between the baseline and after 15-year simulated brushing was approximately 1.5, the resulting color change value was within the perceptibility tolerance of 2.6 Δ*E* [30]. Unlike the previous study that investigated extrinsically characterized zirconia (IPS shade 3), this study assessed the impact of brushing on the color change of intrinsically colored zirconia materials. As extrinsic stains can be easily damaged or removed by external trauma, such as an occlusal reduction procedure, intrinsic coloring is preferred by dentists. However, to date, no study has investigated the impact of brushing on the shade of intrinsically colored zirconia.

In this study, both polished and glazed zirconia specimens exhibited statistically significant color changes; moreover, polished specimens exhibited more color changes than glazed specimens. In addition, polished specimens became considerably darker after toothbrushing, and the color change was at the border of the perceptibility threshold. Furthermore, compared to the polished specimens, the glazed specimens revealed less shade change, which is consistent with Garza et al. [18], who reported that after 12 years of simulated brushing, lithium disilicate specimens glazed after staining were more resistant to color change than specimens that underwent staining and glazing simultaneously. In addition, Alp et al. [31] demonstrated that polished glass ceramics were more susceptible to staining by coffee thermocycling than glazed specimens, suggesting that the glazing layer coated over the colored specimen could play a protective role.

Regarding the surface gloss, Sen et al. [32] reported a marked decline in the gloss of computer-aided design and computer-aided manufacturing (CAD/CAM) restorative materials after 1 year of simulated brushing. This study demonstrated that brushing with fluoride or whitening dentifrices markedly reduced the gloss of zirconia specimens. Moreover, the fluoride-free conventional dentifrice decreased the gloss; however, the difference was not statistically significant. It is possible that the fluoride content or higher RDA of the dentifrice could affect the surface characteristics of zirconia specimens. To date, several studies have demonstrated that acidic and alkaline environments could affect the optical or surface properties of ceramic specimens [13,14,15]. Furthermore, dentin wear is more strongly impacted by the RDA value of dentifrices than by the stiffness of a toothbrush [10].

In this study, the surface roughness of glazed specimens was also marginally affected by use of the fluoride dentifrice. The roughness of zirconia is crucial because it increases the contact area with moisture, which could result in low-temperature degradation [33,34]. In addition, a whitening dentifrice reduced the surface roughness of the polished zirconia surface, which corroborated the results from Pinelli et al. [12]. The high RDA of a whitening dentifrice was considered to exert a polishing effect on the zirconia surface. The glazed groups revealed a rougher surface than the polished groups. Reportedly, the surface roughness threshold for bacterial colonization was 0.2 μm [35,36], and the roughness threshold detectable by the tongue was 0.25–0.5 μm [37]. Although the roughness of the brushed, polished zirconia was within these thresholds, glazed zirconia after brushing exhibited higher results. Furthermore, the results of XRD exhibited no apparent evidence of phase transformation. It is possible that the several high peaks observed in the toothbrushed glazed groups imply partial wear of the glazing layer compared with GDW.

The strength of this study is that the two finishing methods of monolithic zirconia—polishing and glazing—were compared after simulated brushing. In addition, a thick glazing layer could result in errors in the intensity of contact or occlusion of the restoration when fabricated with a modeless CAD/CAM technique; thus, glazing should be selected only when needed. Moreover, brushing was simulated for 8.5 and 17 years, and the long-term effects of brushing were investigated. Previous studies have simulated shorter brushing periods of 1 to 15 years [12,18,30,32]. It is also advantageous to evaluate the number of tooth surfaces to be brushed by the scientific method and calculate the appropriate simulation time and appropriate toothbrush replacement cycle. In addition, three toothpaste formulas were compared in this study. The effects of fluoride were compared by selecting the same brand of fluoride-free toothpaste and high fluoride-containing toothpaste. In addition, a whitening toothpaste with extremely high RDA was also compared. Furthermore, substantial data on various optical and surface properties were obtained through the appropriate arrangement of measurements.

This study has some limitations. The first limitation is the in vitro design. Second, the effects of the aging of zirconia on moisture in the mouth and the fatigue of the material could not be considered because of the accumulated mastication in the clinical setting. Third, DW was used to prepare toothpaste slurry; however, the effect of the mixture of the oral saliva and toothpaste was not investigated. Finally, during the roughness measurement process through a confocal scanning laser system, the voids on the surface of the glazing layer generated in the glazing process were disturbed. Thus, additional clinical studies are warranted to overcome these limitations and validate the findings of this study.

## 5. Conclusions

This study reveals that brushing with several dentifrices markedly affects the optical properties and surface characteristics of monolithic zirconia finished with polishing or glazing methods. Within the limitations of this in vitro study, the following conclusions were drawn:

1. Statistically significant differences were found in the color change of the monolithic zirconia material groups after 17 years of simulated brushing; however, the changes were within the previously reported clinically acceptable threshold [26,27,28,29]. The translucency parameter showed no significant change.
2. Gloss was significantly lower in the groups that were brushed with fluoride toothpaste and whitening toothpaste than that in the unbrushed group. The surface roughness in the glazed group brushed with the fluoride dentifrice was significantly higher than that in the unbrushed group.
3. Minor differences were observed in XRD among the glazing-finished groups. The glazing layer was slightly worn off with any toothpaste and revealed some ZrO_2_ peaks under the silica layer.

The polished groups had significantly lower color stability after brushing; however, the gloss was higher, and the roughness was lower. There was no significant difference noted in translucency.

## Figures and Tables

**Table 1:** Materials used in this study.

Classification	Brand	Manufacturer	Composition *	Code
Monolithic zirconia	Rainbow Shade Block, A2	Genoss	ZrO_2_, Y_2_O_3_ 4–6%, HfO_2_ ≤ 5%, Al_2_O_3_ ≤ 1%, Other oxides.	
Glaze	Glaze HeraCeram	Heraeus Kulzer	SiO_2_ 64.0–66.0%, Al_2_O_3_ 10.4–11.4%, K_2_O 14.5–15.5%, Na_2_O 4.5–5.5%, Other oxides.	
Conventional dentifrice (Fluoride-free)	Parodontax Classic Fluoridfrei	GlaxoSmithKline	Sodium Bicarbonate, Aqua, Glycerin, Alcohol, Cocamidopropyl Betaine, *Mentha arvensis* Oil, *Mentha piperita* Oil, Xanthan Gum, *Echinacea purpurea* Flower/Leaf/Stem Juice, *Krameria triandra* Extract, Chamomilla Recutita Extract, *Salvia officinalis* Oil, *Commiphora myrrha* Extract, Limonene, Sodium Saccharin, Linalool, CI 77491.	C
Fluoride dentifrice	Parodontax Fluorid	GlaxoSmithKline	Sodium Bicarbonate, Aqua, Glycerin, Alcohol, Cocamidopropyl Betaine, *Mentha arvensis* Oil, *Mentha piperita* Oil, Xanthan Gum, *Echinacea purpurea* Flower/Leaf/Stem Juice, *Krameria triandra* Extract, Sodium Fluoride, Chamomilla Recutita Extract, *Salvia officinalis* Oil, *Commiphora myrrha* Extract, Limonene, Sodium Saccharin, Linalool, CI 77491, Enthalt Natriumfluorid (1400 ppm fluoride).	F
Whitening dentifrice	Crest 3d White Vivid	Procter & Gamble	Water, Sorbitol, Hydrated Silica, Disodium Pyrophosphate, Sodium lauryl sulfate, Flavor, Cellulose Gum, Sodium Hydroxide, Sodium Saccharin, Carbomer, Mica, Titanium Dioxide, Blue 1, Sodium Fluoride 0.243% (1500 ppm fluoride ion).	W

* As disclosed by manufacturers.

**Table 2:** Results of the repeated-measures ANOVA with color change (Δ*E*_00_) and translucency parameter as the dependent variable.

Source	Type III Sum of Squares	*df*	Mean Squares	*F*	*p*
Dependent variable: Color change (Δ*E*_00_)					
Time	9.934	1.715	5.794	336.669 ***	<0.001
Time × Group	2.142	12.002	0.178	10.371 ***	<0.001
Error	2.125	123.448	0.017		
Dependent variable: Translucency parameter					
Time	0.025	2.000	0.012	0.908	0.406
Time × Group	0.047	14.000	0.003	0.243	0.998
Error	1.979	144.000	0.014		

*** *p* < 0.001.

**Table 3:** The mean and standard deviation of color change (Δ*E*_00_) values.

Group	Simulated Brushing Time			
	Between Baseline and after 8.5 Years		Between Baseline and after 17 Years	
	Mean	Standard Deviation	Mean	Standard Deviation
PDW	0.2458 ^a^	0.1083	0.3158 ^a^	0.1184
PC	0.4035 ^a,b^	0.1574	0.7164 ^b^	0.1670
PF	0.509 ^b^	0.1817	0.7498 ^b^	0.2881
PW	0.5857 ^b^	0.1716	0.8106 ^b^	0.1946
GDW	0.1988 ^A^	0.0365	0.1953 ^A^	0.0690
GC	0.253 ^A^	0.0727	0.301 ^A,B^	0.1687
GF	0.2643 ^A^	0.1399	0.3051 ^A,B^	0.1735
GW	0.2785 ^A^	0.1443	0.4846 ^B^	0.1600

PDW, polished surface and storage in distilled water; PC, polished surface and brushed with a conventional dentifrice; PF, polished surface and brushed with a fluoride dentifrice; PW, polished surface and brushed with a whitening dentifrice; GDW, glazed surface and storage in distilled water; GC, glazed surface and brushed with a conventional dentifrice; GF, glazed surface and brushed with a fluoride dentifrice; GW, glazed surface and brushed with a whitening dentifrice. Bonferroni: a < b; A < B. Means with the same superscript in each column are not significantly different from each other based on the Bonferroni test (*p* > 0.05).

**Table 4:** Results of two-way ANOVA with color change, translucency parameter, surface gloss, and surface roughness as the dependent variable.

Source	Type III Sum of Squares	*df*	Mean Squares	*F*	*p*
Dependent variable: Color change (Δ*E*_00_)					
Finishing	2.134	1	2.134	67.84 ***	<0.001
Dentifrice	1.629	3	0.543	17.262 ***	<0.001
Finishing × Dentifrice	0.322	3	0.107	3.407 *	0.022
Error	2.265	72	0.031		
Dependent variable: Translucency parameter					
Finishing	0.060	1	0.060	0.634	0.428
Dentifrice	0.213	3	0.071	0.754	0.524
Finishing × Dentifrice	0.065	3	0.022	0.231	0.874
Error	6.773	72	0.094		
Dependent variable: Gloss (GU)					
Finishing	4124.192	1.000	4124.192	22.886 ***	<0.001
Dentifrice	2477.803	3.000	825.934	4.583 **	0.005
Finishing × Dentifrice	125.081	3.000	41.694	0.231	0.874
Error	12974.882	72	180.2067		
Dependent variable: Roughness (Ra)					
Finishing	4.266	1.000	4.266	97.718 ***	<0.001
Dentifrice	0.363	3.000	0.121	2.769 *	0.048
Finishing × Dentifrice	0.261	3.000	0.087	1.990	0.123
Error	3.143	72	0.044		

*** *p* < 0.001; ** *p* < 0.01; * *p* < 0.05.

**Table 5:** The mean and standard error of each dependent variable according to the finishing methods and dentifrice used.

		Color Change (Δ*E*_00_) *		Translucency Parameter		Gloss (GU)		Roughness (Ra: µm)	
Group	*n*	Mean	Standard Error	Mean	Standard Error	Mean	Standard Error	Mean	Standard Error
Finishing									
P	40	0.6481 ^b^	0.028	4.7545 ^a^	0.0485	96.075 ^b^	2.123	0.132 ^a^	0.033
G	40	0.3215 ^a^	0.028	4.6999 ^a^	0.0485	81.715 ^a^	2.123	0.594 ^b^	0.033
Dentifrice									
DW	20	0.2555 ^A^	0.04	4.7742 ^A^	0.0686	93.8 ^B^	3.002	0.298 ^A^	0.047
C	20	0.5087 ^B^	0.04	4.7819 ^A^	0.0686	93.275 ^B^	3.002	0.321 ^A^	0.047
F	20	0.5275 ^B^	0.04	4.6881 ^A^	0.0686	88.585 ^A,B^	3.002	0.473 ^A^	0.047
W	20	0.6476 ^B^	0.04	4.6647 ^A^	0.0686	79.92 ^A^	3.002	0.36 ^A^	0.047

P, polished; G, glazed; DW, storage in distilled water; C, brushed with a conventional dentifrice; F, brushed with a fluoride dentifrice; W, brushed with a whitening dentifrice. Bonferroni: a < b; A < B. Means with the same superscript in each column are not significantly different from each other based on the Bonferroni test (*p* > 0.05). * Color change (Δ*E*_00_) between the baseline and 17 years of simulated brushing.

**Table 6:** The mean and standard deviation of translucency parameter values.

Group	Simulated Brushing Time					
	Baseline		8.5 Years		17 Years	
	Mean	Standard Deviation	Mean	Standard Deviation	Mean	Standard Deviation
PDW	4.7715	0.1008	4.7717	0.2972	4.7731	0.3186
PC	4.7829	0.2804	4.7802	0.2432	4.7807	0.2615
PF	4.7681	0.3191	4.7535	0.2811	4.7464	0.3464
PW	4.7585	0.4046	4.7484	0.4712	4.7179	0.4237
GDW	4.7675	0.3011	4.7696	0.2563	4.7753	0.2633
GC	4.7851	0.3517	4.7830	0.3311	4.7831	0.2908
GF	4.7297	0.2942	4.6916	0.2870	4.6297	0.2552
GW	4.6526	0.2499	4.6406	0.2856	4.6115	0.2533

PDW, polished surface and storage in distilled water; PC, polished surface and brushed with a conventional dentifrice; PF, polished surface and brushed with a fluoride dentifrice; PW, polished surface and brushed with a whitening dentifrice; GDW, glazed surface and storage in distilled water; GC, glazed surface and brushed with a conventional dentifrice; GF, glazed surface and brushed with a fluoride dentifrice; GW, glazed surface and brushed with a whitening dentifrice. No significant difference was shown (*p* > 0.05).

**Table 7:** The mean and standard deviation of surface gloss (GU) and surface roughness (Ra) values.

Group	*n*	Gloss (GU)		Roughness (Ra: µm)	
		Mean	Standard Deviation	Mean	Standard Deviation
PDW	10	102.4 ^a^	19.98	0.1549 ^a^	0.0911
PC	10	101.33 ^a^	14.68	0.0976 ^a^	0.0735
PF	10	93.97 ^a^	19.32	0.1759 ^a^	0.1097
PW	10	86.6 ^a^	20.14	0.1004 ^a^	0.0507
GDW	10	85.2 ^A^	1.55	0.441 ^A^	0.1614
GC	10	85.22 ^A^	1.13	0.5443 ^A,B^	0.2540
GF	10	83.2 ^A^	2.99	0.7704 ^B^	0.2819
GW	10	73.24 ^A^	5.98	0.6205 ^A,B^	0.3885

PDW, polished surface and storage in distilled water; PC, polished surface and brushed with a conventional dentifrice; PF, polished surface and brushed with a fluoride dentifrice; PW, polished surface and brushed with a whitening dentifrice; GDW, glazed surface and storage in distilled water; GC, glazed surface and brushed with a conventional dentifrice; GF, glazed surface and brushed with a fluoride dentifrice; GW, glazed surface and brushed with a whitening dentifrice. Bonferroni: A < B. Means with the same superscript in each column are not significantly different from each other based on the Bonferroni test (*p* > 0.05).
